# Lipopolysaccharide Enhances Genotoxicity by Activating GADD45G and NF-*κ*B in Human Corneal Epithelial Cells

**DOI:** 10.1155/2022/4328116

**Published:** 2022-01-04

**Authors:** Ramachandran Samivel, Umadevi Subramanian, Adnan Ali Khan, Omar Kirat, Ali Masmali, Turki Almubrad, Saeed Akhtar

**Affiliations:** ^1^Cornea Research Chair, Department of Optometry, College of Applied Medical Sciences, King Saud University, Saudi Arabia; ^2^Translational Research Platform for Veterinary Biologicals, Central University Laboratory Building, TANUVAS, Tamil Nadu, India; ^3^Department of Ophthalmology, King Khalid Eye Specialist Hospital, Riyadh, Saudi Arabia; ^4^College of Applied Medical Science, Inaya Medical College, Riyadh, Saudi Arabia

## Abstract

As the prevalence of microbial keratitis increases, it creates an environment conducive to genotoxicity response. A potential connection between growth arrest and DNA-damage-inducible 45 gamma (GADD45G) gene expression has not been proven in the corneal epithelial cells. The aim of this study was to determine whether lipopolysaccharide (LPS) enhances genotoxicity, DNA damage, and inflammatory responses in human corneal epithelial cells (HCECs) *in vitro*. In a set of parameters, cytotoxicity, reactive oxygen species, mitochondrial membrane potential, DNA damage, inflammatory response, and apoptosis were assessed. LPS (1, 5, and 10 *μ*g/mL) treated HCECs were increased reactive oxygen species formation, mitochondrial membrane depolarization, and genotoxicity in a concentration-dependent manner. Similarly, NF-*κ*B, PARP1, and TP53 were also overexpressed in the LPS treated HCECs. 24 hours after LPS induction, micronucleus scoring, and proapoptotic factors were also increased. Among them, the GADD45G, NF-*κ*B, and *γ*H2AX were overexpressed both on the mRNA and protein levels in LPS (10 *μ*g/mL) treated HCECs. In our study, we show that the GADD45G signaling can trigger genotoxic instability in HCECs exposed to LPS. Therefore, understanding the factors contributing to infectious keratitis, such as GADD45G, NF-*κ*B, and *γ*H2AX signaling, may help to develop antigenotoxic and anti-inflammatory therapies for corneal dystrophy and epithelial cell remodeling.

## 1. Introduction

Corneoscleral interface of the limbal epithelium serves as a barrier that protects the eye from bacterial invasion and serves as a source of stem cells [1]. The cornea is affected by a microbial infection that manifests as corneal inflammations, corneal thinning, and ultimately corneal perforation and scarring [2, 3]. It is characterized by the presence of diffuse or localized infiltrates on the anterior surface of the ocular tissues and corneal epithelium. The epithelium and stroma of patients with severe keratitis exhibit necrotic or apoptotic ulceration. Often, the late stage of bacterial keratitis can be recognized by the absence of epithelium and the presence of suppurative stromal infiltrates [4, 5]. There is an increasing incidence of microbial infectious diseases; contact lens wearers make up one of the biggest risk factors for causing infectious keratitis [6]. Numerous epidemiological studies have consistently indicated that overnight contact lens wear also increases the incidence of microbial keratitis [7–9].

Gram-negative bacteria produce lipopolysaccharide (LPS) as a major component of their outer membrane. It greatly contributes to the structural integrity of the bacterial cell wall. LPS is a well-known endotoxin and has proven effective for inducing inflammation in corneal dystrophy models [10–14]. The LPS-induced keratitis can rapidly progress to infectious ocular disease, which is one of the most common causes of corneal dystrophy. However, a number of key factors were found responsible for the pathogenesis of LPS-induced cellular injury, such as oxidative stress, cytotoxicity, and inflammatory response [15, 16]. Human corneal epithelial cells (HCECs) provide a new opportunity for testing *in vitro* ocular and corneal toxicity. As the primary corneal epithelial cells stop growing after 5-6 passages, their proliferation is limited [17]. Specifically, the current study is aimed at examining the DNA damage and genomic instability caused by exposure to LPS in human corneal epithelial cells. Several previous studies have shown that frequent exposure to endotoxins leads to DNA damage that is critical for maintaining cellular homeostasis [18–20]. Upon insufficient DNA damage repair, point mutations, gene transcriptional changes, and function deficits can occur and trigger the activation of signal transducers and epigenetic modifications [13].

Among the oxidative stress-related genes, the member of the Growth arrest and DNA-damage-inducible 45 gene family (GADD45) possessed three isotype GADD proteins, GADD45*α*, *β*, and *γ*. These proteins are acidic, have pleiotropic effects, and are found in the nucleus of the cell. Particularly, GADD45 (GRP17/CR6) family proteins share 55% to 58% amino acid homology and mediate the activation of C-Jun NH2-terminal kinase in response to multiple environmental stresses [21]. Genotoxic instability and oxidative stress can result in DNA damage, cell cycle arrest, apoptosis, and epigenetic modifications, resulting in an increase in cell survival and/or senescence. Apoptosis can also be induced by them at the G1-S and G2-M checkpoints, or they can inhibit cell proliferation at different stages of the cell cycle arrest [22]. The expression of GADD45*γ* also stimulates the DNA damage response through the generation of ROS and the formation of aberrant DNA replication intermediates, which may trigger the activation of H2AX and its cofactors. This may trigger the activation of H2AX phosphorylation and DNA fragmentation in a single cell [23]. Based on these observations, the present study attempted to determine whether LPS contributes to genotoxic instability and participates in GADD45*γ* mediated phosphor *γ*H2AX formation and induction of apoptosis in the HCECs *in vitro* model.

## 2. Materials and Methods

### 2.1. Human Corneal Epithelial Cell Culture

The complete corneal rims were procured from Timestrip Plus (Oklahoma Lions Eye Bank, LN, USA). The corneoscleral rims were obtained from the surgeon at King Khaled Eye Specialist Hospital, Riyadh, Saudi Arabia, following corneal transplantation. During the procurement and use of human tissue, the Declarations of Helsinki were followed strictly. The ethics committees at both King Saud University and King Khaled Eye Specialist Hospital (Riyadh, Saudi Arabia) approved the efficacy and safety of the study. Isolation of human corneal epithelial cells (HCECs) from these tissues has been described previously [24]. A brief description of the procedure is as follows: corneoscleral rim tissues were incubated overnight with 2.4 IU dispase II (Cat No. 42613-33-2; Sigma-Aldrich, USA) in HEPES buffer. The epithelial layer was removed from the limbal area and seeded with 5 × 10^5^ cells/cm^2^ in 60 mm Petri plates (Corning Inc., Corning, NY, USA), incubated with Dulbecco's modified Eagle's medium and Ham's F12 medium (1 : 1), and additional supplements 1× ITS (Insulin: Transferrin: Sodium selenite) mixture, 10% fetal bovine serum, 10 ng/mL epidermal growth factor (EGF), 100 ng/mL hydrocortisone, 25 *μ*M HEPES, and 1% antibiotic-antimycotic solutions at 37°C in an atmosphere with 5% CO_2_. Once the HCEC cells reached 80% confluence, they were subcultured under the same conditions used for all subsequent experiments.

### 2.2. Cell Viability Assay

The viability of the cells was assessed with a colorimetric assay using 3-(4,5-dimethylthiazol-2-yl)-2,5 diphenyl tetrazolium bromide (MTT) as described previously [25]. In brief, HCEC cells (1 × 10^4^ cells/well) were plated in 96-well plates overnight and treated with LPS at concentrations ranging from 1–70 *μ*g/mL. Added 20 *μ*L of 5 g/L MTT (Sigma-Aldrich) to each well and incubated the plates for 4 hours in the dark at 37°C after 24, 48, and 72 hours of incubation with the HCECs. Afterward, 200 *μ*L of dimethyl sulfoxide (Sigma-Aldrich) was added to each well to dissolve the generated formazan crystals. Based on HCEC viability, the formazan color substance was measured using a multimode microplate reader at 490 nm (Tecan Infinite 200, Switzerland).

### 2.3. Assessment of Intracellular Reactive Oxygen Species (ROS) Generation

In the cells exposed to the oxidative stress caused by LPS induction, the amount of intracellular ROS was measured using 2′, 7′-diacetyl dichlorofluorescein diacetate (DCFH-DA) [26]. In brief, HCECs (5 × 10^5^) were seeded in a 6-well plate and treated with varying concentrations of LPS (0, 1, 5, and 10 *μ*g/mL). The HCECs were harvested after 24 hours of induction and suspended in phosphate-buffered saline (PBS). 10 *μ*M/mL DCFH-DA solutions were added and incubated at 37°C in 5% CO_2_ for 15 minutes. Cells were washed three times with FACS buffer. Cell number was counted by BD Accuri C6 flow cytometer (BD Bioscience, USA), and images were captured at 488/525 nm using the Olympus BX53 fluorescence microscope (Olympus Inc., Melville, NY, USA).

### 2.4. Quantification of the Mitochondrial Membrane Potential

A previously described method was used to determine the mitochondrial membrane potential [27]. Briefly, HCECs (5 × 10^5^) were seeded in 6-well plates and treated with varying concentrations of LPS (0, 1, 5, and 10 *μ*g/mL). In the following 24 hours, the HCECs were harvested and treated with 2.5 *μ*M/mL of JC-1 (5,5′ 6,6′-tetrachloro-1,1′3,3′-tetraethyl-imidacarbocyanine iodide, Sigma Aldrich) dye. For 30 minutes, the cells were incubated in a humidified atmosphere with 5% CO_2_ at 37°C in the dark. Cell pellets were washed and centrifuged and then suspended in FACS buffer. The cells were counted on a BD Accuri C6 flow cytometer (BD Bioscience, USA), and the images were captured on an Olympus BX53 fluorescence microscope (Olympus Inc., Melville, NY, USA).

### 2.5. Assessment of Cytokinesis-Blocked Micronucleus (CBMN) Assay

CBMN assay was performed using cytochalasin B, as described in the previous protocol [28], with minor modifications. In brief, HCECs (5 × 10^5^) were seeded in 6-well plates and treated with varying concentrations of LPS (0, 1, 5, and 10 *μ*g/mL). HCECs were washed with PBS and incubated with serum-free medium containing cytochalasin B (final concentration 6 *μ*g/mL) at 37°C in a humidified atmosphere with 5% CO_2_ for 48 hours. The cells were followed by washing with PBS, harvesting with hypotonic solution (0.56% KCl) at 4°C for 3 minutes, fixing with ice-cold Carnoy's fixative (mixture of methanol and glacial acetic acid, 3 : 1 ratio), and air-dried on coded microscopic glass slides. After the slides had been stained with 4% Giemsa solution for 8 minutes, they were completely rinsed and placed to dry at 37°C. For each experiment, a total number of 1000 binucleated cells were scored blind at a 60× magnification.

### 2.6. Apoptosis DAPI/PI Staining

DAPI/propidium iodide (PI) staining was carried out using a previously described method, with slight modifications [29]. A brief overview of the experiment is that the HCECs were grown in sterile glass coverslips under 12-well plates for overnight and treated with different concentrations of LPS (0, 1, 5, and 10 *μ*g/mL). Cells were incubated for 24 hours in a humidified atmosphere containing 5% CO_2_ at 37°C. After incubation, the cells were washed with PBS and fixed in 3.7% paraformaldehyde in PBS pH 7.2 for 15 minutes at 37°C. Cells were stained with DAPI and PI in PBS (2 *μ*g/mL each) and incubated for 15 minutes in the dark. After that washed the cells in PBS and analyzed the morphology with blue channel (358/461) and red channel (538/617) fluorescence microscopy (BX53 Olympus, Germany) to score the apoptotic bodies.

### 2.7. Analysis of mRNA Expressions by qRT-PCR

The total RNA was extracted with TRIzol (Invitrogen, Carlsbad, CA, USA) following the manufacturer's instructions. Using the iScript cDNA synthesis kit (Bio-Rad, KSA), equivalent amounts of total RNA (1 *μ*g/sample) were reverse-transcribed. qRT-PCR was performed using a real-time PCR system from Applied Biosystems® 7500 FAST (CA, USA). The sources of primers were listed in Table 1. The reactions were conducted in a 20 *μ*L reaction mixture using the Qiagen RT^2^ SYBR Green qPCR Mastermix (California, USA). During the reaction, 95°C was applied for 10 minutes, followed by 40 cycles of 30 seconds at 95°C and 1 minute at 60°C. We used the reaction mixture without the template cDNA as a negative control. mRNA expression was normalized to that of endogenous controls (GAPDH; glyceraldehydes 3-phosphate dehydrogenase) within each sample. A relative gene expression analysis was conducted using the 2^-*ΔΔ*CT^ method.

### 2.8. Analysis of mRNA Expressions by RT-PCR

Reverse transcriptase PCR was performed using a thermocycler (Applied Biosystems, CA, USA). The 25 *μ*L reaction mixtures are made up of 2 *μ*L cDNA, 2 *μ*L forward and reverse primers (Table 1), and 12.5 *μ*L 2x PCR Mastermix. Annealing was performed at 55°C for 40 cycles. Amplification was completed by heating the reaction mixture to 72°C for 10 minutes and cooling it to 4°C. A 10 *μ*L sample of each PCR product was separated using 2% Agarose gel electrophoresis containing 0.5 *μ*g/mL ethidium bromide. DNA molecular length markers were used to analyze the agarose gel under ultraviolet light. The signal was detected and quantified using the Alpha Innotech imaging system (San Leandro, CA, USA).

### 2.9. Western Blot Analysis

HCECs (5 × 10^6^) treated for 24 hours with LPS (10 *μ*g/mL) were lysed for protein expression by Western blotting analysis as described previously [30]. The cells were washed in PBS and harvested with a cell scraper. After resuspending the cell pellets in 500 *μ*L of ice-cold cell lysis buffer with 1× Proteinase inhibitor cocktails (Sigma-Aldrich), the buffer was incubated at 4°C for 1 hour. Cell suspensions were sonicated five times for 30 seconds each and then centrifuged at 12,000 g for 30 minutes at 4°C. Total protein was quantified using a BCA kit (Pierce, Thermo Scientific, Madison, WI, USA). Cell extracts (30 *μ*g of protein/well) were mixed with 4× sample loading buffer, and 12% sodium dodecyl sulfate-polyacrylamide gel electrophoresis (SDS-PAGE) was used to separate the proteins under reducing conditions. Trans-Blot (Turbo, Bio-Rad) at 25 V for 30 minutes was used to transfer gels to PVDF membrane. A solution of 1× Tris-buffered saline/Tween 20 (TBST) containing 5% nonfat milk powder was used to block the membrane for 1 hour at room temperature. The membranes were incubated overnight at 4°C with specific primary antibodies (Table 2) diluted to 1 : 1000 in TBST containing 5% nonfat milk powder. Following three washes in TBST for 5 minutes each, the membrane was then incubated with 1 : 2000 horseradish peroxidase-conjugated secondary antibodies for 1 hour. The membrane was washed three times with TBST and developed using SuperSignal West Femto Chemiluminescent Substrate (Thermo Scientific, Madison, WI, USA). We detected chemiluminescent signal with the CDigit scanning system (Riyadh, Saudi Arabia).

### 2.10. Immunohistochemical Staining

HCECs were immunostained with DAB-HRP complex staining (3,3′-diaminobenzidine tetrahydrochloride kit) following the manufacturer's instructions, with minor modifications. HCECs (5 × 10^4^) were grown in sterile glass slides under 12-well plates overnight and treated with various concentrations of LPS (0, 1, 5, and 10 *μ*g/mL). Cells were incubated for 24 hours in 5% CO_2_ at 37°C in humidified atmosphere. Following incubation, cells were washed with PBS, fixed with 3.7% paraformaldehyde in PBS pH 7.2 for 30 minutes, and then permeabilized with 0.1% Triton X-100 in PBS for 10 minutes. Blocked nonspecific binding using 3% normal goat serum and incubated the slides with a primary polyclonal antibody (1 : 200) diluted in 1% BSA diluted in PBS with 0.1% Tween-20 (PBST) for 2 hours at 37°C. Slides were washed three times with PBST before and after incubation with goat anti-rabbit and goat anti-mouse HRP-conjugated secondary antibodies (1 : 500) for 1 hour at 37°C. Afterward, the peroxidase activity was determined by treating them with the 3,3′-diaminobenzidine tetrahydrochloride kit (Sigma-Aldrich, Darmstadt, Germany). Meyer's hematoxylin was counterstained according to the manufacturer's instructions. At the end, the slides were dehydrated and covered with Aquatex® (HC440258, Merck, Germany) mounting medium. The cells with positive reactivity to the proteins were determined using a blinded quantitative analysis performed with Image-J analysis software under a light microscope. The number of positive cells per 1000 cells was counted for each section under high magnification (20×).

### 2.11. Statistical Analysis

The results were expressed as Mean ± Standard Error. The significance of the results was evaluated using one-way analysis of variance, followed by Dunnett's multiple comparison tests using GraphPad Prism Software version 8 (GraphPad Software, San Diego, CA, USA). The *P* values of 0.05, 0.01, and 0.001 represent ∗, ∗∗, and ∗∗∗ vs. normal control cells (NC).

## 3. Results

### 3.1. Detection of Cell Viability in HCECs Treated with LPS

**Figure 1:**
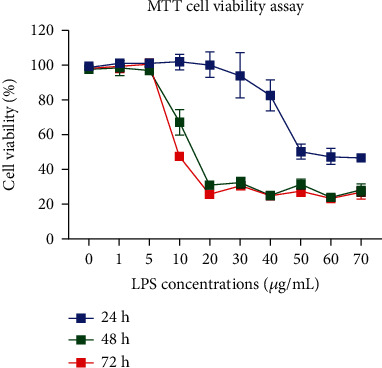
Measurement of cell viability in lipopolysaccharide-treated HCEC cells. In a LPS concentration and time-dependent manner, cytotoxicity was increased in HCECs. This analysis used three cultures replicates per experimental point (*n* = 6), and the statistical test used *P* values of 0.05, 0.01, and 0.001 represent ∗, ∗∗, and ∗∗∗ compared to untreated control cells (NC) using Kruskal-Wallis and Dunn's tests.

The viability of HCECs treated with LPS was evaluated at 24, 48, and 72 hours (Figure 1). During the 24 hours LPS treatment, the cell viability was reduced at high concentrations (30-70 *μ*g/mL), but not at lower concentrations (0-20 *μ*g/mL). At both 48 and 72 hours, LPS-treated HCECs at concentrations ranging from 10 *μ*g/mL to 70 *μ*g/mL decreased viability compared to the untreated control. In contrast, the lower concentrations of 0-5 *μ*g/mL did not affect the viability of the cells. As a result, we used three intermediate concentrations of LPS (1, 5, and 10 *μ*g/mL) in HCECs for further cell and molecular analysis.

### 3.2. Analysis of Intracellular ROS in HCECs Treated with LPS

**Figure 2:**
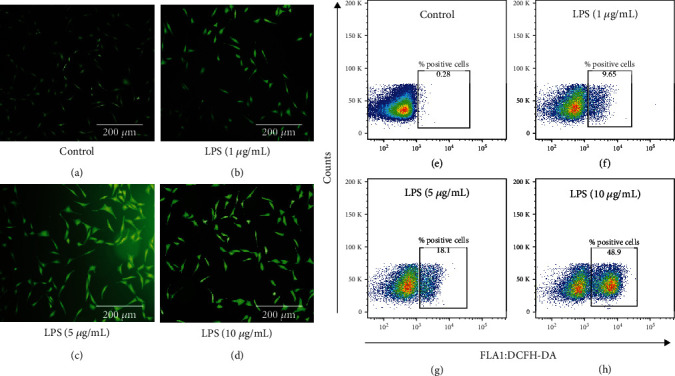
Analysis of intracellular ROS accumulation in lipopolysaccharide-treated HCEC cells. Fluorescence microscopy and flow cytometry were used to measure ROS elevations in HCEC cells. DCFH-stained positive HCEC cells were increased in LPS 1, 5, and 10 *μ*g/mL concentrations after 24 hours of incubation (a)–(d). The percentage of positive cells in the LPS treated HCECs is shown in the results of the FACS analysis (e)–(h). Based on three cultures replicated for each experimental points (*n* = 6), the data represent Mean ± Standard Error (S.E.).

Fluorescence microscopy was used to determine the intracellular ROS levels in HCECs treated with LPS (Figure 2). In untreated control HCECs, DCFH showed weak fluorescent (Figure 2A). At 24 hours after treatment with LPS (1, 5, and 10 *μ*g/mL), a gradual increase in DCFH fluorescence was observed in Figures 2(b)–2(d). It indicates that ROS levels were increased in HCECs treated with LPS. As a result of the flow cytometry analysis results, only 0.25% of DCFH stained positive cells were gated in untreated control HCECs (Figure 2(e)). As shown in Figures 2(f)–2(h), DCFH-stained positive cell population gated HCECs were increased by 9.65 ± 0.40, 18.1 ± 1.22, and 48.9 ± 2.80% in LPS treatment (1, 5, and 10 *μ*g/mL) at 24 hours, respectively.

### 3.3. Measurement of the Mitochondrial Membrane Potential in HCECs Treated with LPS

**Figure 3:**
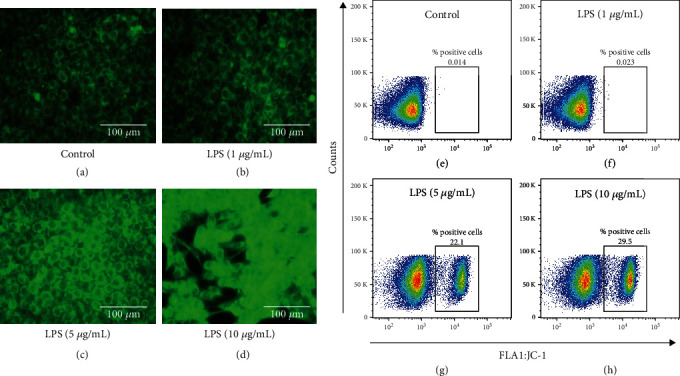
Assessment of the mitochondrial membrane potential in HCEC cells treated with lipopolysaccharide (LPS). Fluorescence microscopy and flow cytometry were used to analyze the staining of HCEC cells with JC-1 dye. Incubation of LPS (1, 5, and 10 *μ*g/mL) for 24 hours (a)–(d) shows bright green fluorescent staining in the HCEC cells. A FACS analysis shows the percentage of positive cells in the LPS-treated HCECs (e)–(h). Based on three cultures replicated for each experimental points (*n* = 6), the data represent Mean ± Standard Error (S.E.).

The mitochondrial membrane potential in HCECs treated with LPS was measured by fluorescence microscopic images (Figure 3). A weak fluorescent staining was observed in the untreated control HCECs, and the cells were treated with 1 *μ*g/mL LPS (Figure 3(a) and 3(b)). On contrary, bright green fluorescent staining was visualized in cells treated with 5 and 10 *μ*g/mL LPS (Figures 3(c) and 3(d)), indicating that mitochondrial depolarization was increased in HCECs treated with LPS for 24-hour incubation. The flow cytometry analysis was carried out to analyze the mitochondrial tracking dye JC-1 in HCECs treated with LPS. The JC-1 stained positive cell population was observed in 0.014 and 0.023% in the untreated and treated HCECs with 1 *μ*g/mL of LPS, respectively (Figures 3(e) and 3(f)). On the other hand, JC-1 stains positive cell population gated HCEC cells were increased (22.1 ± 1.10 and 29.5 ± 1.24%) with the LPS treatment (5 and 10 *μ*g/mL) at 24-hour incubation (Figures 3(g) and 3(h)), respectively.

### 3.4. Assessment of Micronuclei Formation in HCECs Treated with LPS

**Figure 4:**
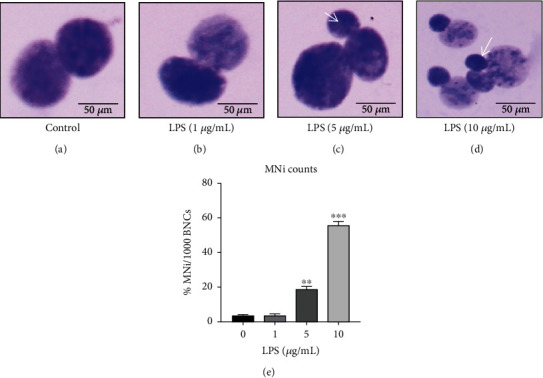
Assessment of micronuclei formation in lipopolysaccharide- (LPS-) treated HCECs. The light microscopic images of micronuclei formed in HCEC cells treated with LPS (1, 5, and 10 *μ*g/mL) are illustrated in panels (a)–(d). The light microscopic image quantitative counts in HCEC cells show in the graph (e). Based on three cultures replicated for each experimental points (*n* = 6), the data represent Mean ± Standard Error (S.E.). *P* values of 0.05, 0.01, and 0.001 representing ∗, ∗∗, and ∗∗∗ as compared to untreated control cells (NC) were calculated using Kruskal-Wallis and Dunn's tests. The images were all captured at a 60× magnification.

Light microscopic images of HCECs treated with LPS were used to quantify the number of micronuclei formed (Figure 4). The untreated control cells and HCECs treated with LPS (1 *μ*g/mL) did not exhibit micronuclei in the nucleus during mitotic induction (Figures 4(a) and 4(b)). HCECs treated with LPS at 5 and 10 *μ*g/mL showed fragmented micronuclei frequencies (Figures 4(c) and 4(d)), which were calculated to be 18.22 ± 0.56 and 58.65 ± 2.47%, respectively, in mitotic induction (Figure 4(e)).

### 3.5. Assessment of Apoptotic Induction in HCECs Treated with LPS

**Figure 5:**
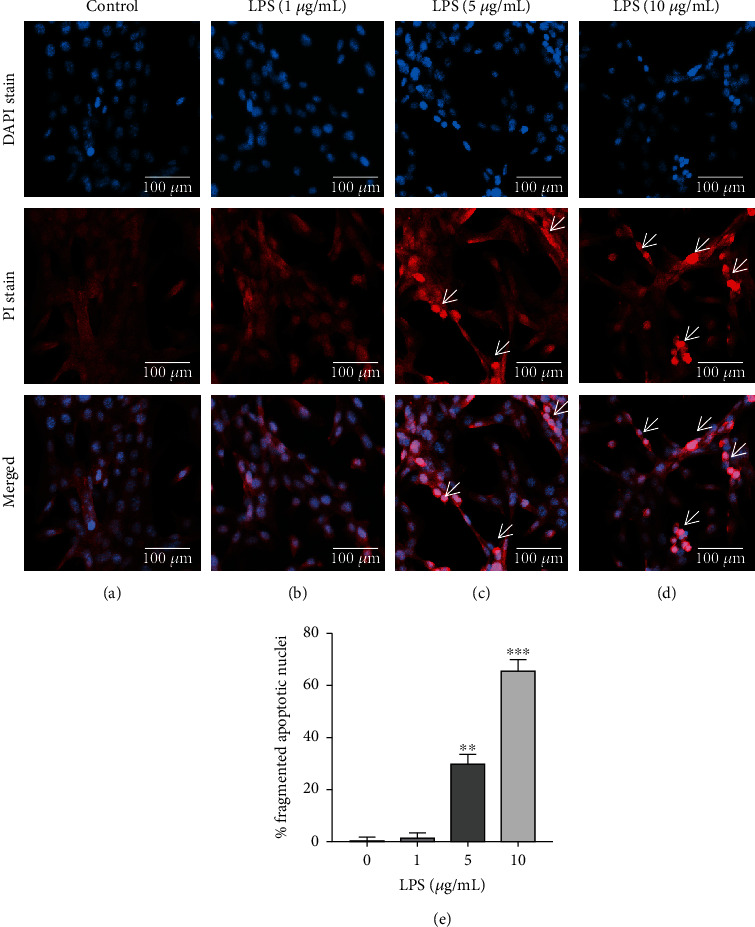
An analysis of fluorescent microscopic images of apoptosis induction in HCEC cells treated with lipopolysaccharide (LPS). DAPI/PI staining in HCEC cells treated with LPS (1, 5, and 10 *μ*g/mL) at 24 hours is illustrated in panels (a)–(d). The microscopic image quantitative counts in HCEC cells show in the graph (e). Based on three cultures replicated for each experimental points (*n* = 6), the data represent Mean ± Standard Error (S.E.). *P* values of 0.05, 0.01, and 0.001 representing ∗, ∗∗, and ∗∗∗ as compared to untreated control cells (NC) were calculated using Kruskal-Wallis and Dunn's tests. The images were all captured at a 40× magnification.

By fluorescence microscopy, the apoptotic induction of HCECs treated with LPS was observed (Figure 5). Untreated control cells and HCECs treated with LPS (1 *μ*g/mL) did not show any DNA condensation or fragmentation in Figures 5(a) and 5(b). The HCECs treated with LPS at 5 and 10 *μ*g/mL concentrations showed apoptotic fragmented bodies as indicated by white arrows in Figures 5(c) and 5(d). Quantified data were presented as a percentage of apoptosis (Figure 5(e)). These levels were increased by 35.87 ± 1.82 and 69.28 ± 2.34% in HCECs treated with LPS (5 and 10 *μ*g/mL) after 24 hours of incubation, respectively.

### 3.6. Assessment of Gene Expression in HCECs Treated with LPS

**Figure 6:**
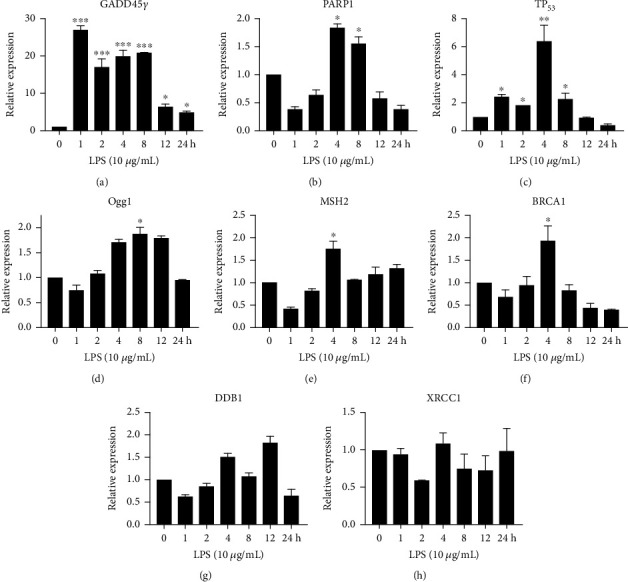
An analysis mRNA expression profiles of DNA damage, growth arrest, and DNA repair genes in HCECs treated with lipopolysaccharide (LPS). The following qRT-PCR experiments were carried out using LPS treated HCEC cells to examine mRNA levels for GADD45G, PARP1, Tp53, MSH2, OGG1, BRCA1, DDB1, and XRCC1 (a)–(h). Quantification of the mRNA expression was performed at time points of 0, 1, 2, 4, 8, 12, and 24 hours. Based on three cultures replicated for each experimental points (*n* = 6), the data represent Mean ± Standard Error (S.E.). *P* values of 0.05, 0.01, and 0.001 representing ∗, ∗∗, and ∗∗∗ as compared to untreated control cells (NC) were calculated using Kruskal-Wallis and Dunn's tests.

The expression profiles of oxidative stress-induced DNA damage-inducible, cell cycle arrest, and DNA repair genes were determined by qRT-PCR in untreated HCECs and HCECs treated with LPS (10 *μ*g/mL) (Figure 6). In LPS treated HCECs, GADD45G expression was highly increased for 8 hours (*P* < 0.001) and then decreased for 12 and 24 hours compared with untreated control cells (Figure 6(a)). HCECs were found to express PARP1 gene at 4 and 8 hours after LPS treatment, and this gene expression decreased at 12 and 24 hours (*P* < 0.05) compared with control cells (Figure 6(b)). The expression of the proapoptotic marker p53 was higher at 4 hours, but decreased until 24 hours in LPS-treated HCECs (*P* < 0.01) compared with the control cells (Figure 6(c)). The expression of genes involved in DNA repair and growth arrest (MSH2, Ogg1, DDB1, XRCC1, and BRCA1) was slightly increased in LPS treated HCECs but did not differ significantly from that in the untreated control cells (Figures 6(d)–6(h)).

**Figure 7:**
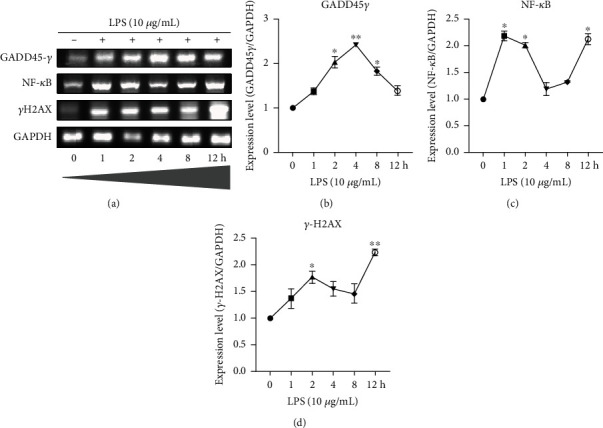
The expression of GADD45G, NF-*κ*B, and H2AX by RT-PCR in HCEC cells treated with lipopolysaccharide (LPS). mRNA expression levels of HCEC cells treated with LPS at 0, 1, 2, 4, 8, and 12 h were quantified (a)–(d). Relative expression and density of the target gene were normalized to that of housekeeping gene GAPDH. Based on three cultures replicated for each experimental points (*n* = 6), the data represent Mean ± Standard Error (S.E.). *P* values of 0.05, 0.01, and 0.001 representing ∗, ∗∗, and ∗∗∗ as compared to untreated control cells (NC) were calculated using Kruskal-Wallis and Dunn's tests.

Through RT-PCR, the mRNA expression of GADD45G, NF-*κ*B, and *γ*H2AX was examined in untreated control and LPS 10 *μ*g/mL treated HCECs (Figure 7). At 4 hours, LPS-treated HCECs exhibited increased GADD45G expression (*P* < 0.01), whereas at 12 hours, it decreased relative to the untreated control (Figures 7(a) and 7(b)). The expression of the NF-*κ*B gene was increased in LPS-treated HCECs at 1, 2, and 12 hours (*P* < 0.05) and decreased at 4 and 8 hours, compared with untreated controls (Figure 7(c)). Similarly, compared to untreated control HCECs, the *γ*H2AX expression was linearly increased at a time interval of 1-8 hours, after which it was highly elevated at 12 hours (*P* < 0.01) (Figure 7(d)).

### 3.7. Analysis of Protein Expressions in HCECs Treated with LPS

**Figure 8:**
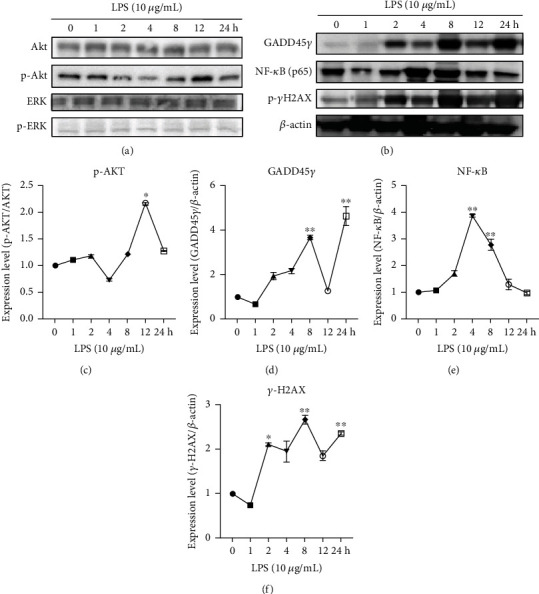
In lipopolysaccharide- (LPS-) treated HCECs, survival growth factor, growth arrest, and DNA damage-inducible protein expression have been examined. Western blots showing photographic images of cell Akt and Erk (a). An immunoblot showing the Gadd45*γ*, pNF-*κ*B (p65), and phosphor *γ*H2AX protein expression (b). In (c), the graph shows the levels of Akt, p-Akt, Erk, and p-Erk protein expression, which were determined by densitometry analysis using the Image-J software. The graph (d)–(f) show the expressions of the Gadd45*γ*, pNF-*κ*B (p65), and phosphor *γ*H2AX. These expressions are quantified by densitometric analysis using Image-J software. The relative density bands of the target gene were normalized to expression of the housekeeping gene *β*-actin. Based on three cultures replicated for each experimental points (*n* = 6), the data represent Mean ± Standard Error (S.E.). *P* values of 0.05, 0.01, and 0.001 representing ∗, ∗∗, and ∗∗∗ as compared to untreated control cells (NC) were calculated using Kruskal-Wallis and Dunn's tests.

Western blotting (Akt, Erk, Gadd45*γ*, pNF-*κ*B (p65), and *γ*H2AX) was performed on HCECs at various time points treated with LPS 10 *μ*g/mL and untreated control cells (Figures 8(a) and 8(b)). In LPS treated HCECs, the expression of p-Akt protein was decreased until 4 hours, but after that, phosphorylated Akt levels had increased at 8 and 12 hours (Figure 8(c)), but Erk and p-Erk did not change. After 8 and 24 hours of LPS treatment, the protein expression of Gadd45*γ* was greatly increased in HCECs (*P* < 0.01), compared with untreated control cells (Figure 8(d)). The phosphor-NF-*κ*B (p65) nuclear protein was significantly elevated in LPS treated HCECs at 4 and 8 hours and then decreased at 12 and 24 hours (*P* < 0.01) compared to the untreated control cells (Figure 8(e)). In LPS-treated HCECs, the DNA double-strand breaking marker phosphor-*γ*H2AX expression is linearly increased at 2–24 hours, but its levels are significantly elevated at 8 hours (*P* < 0.01) compared with untreated control cells (Figure 8(f)).

### 3.8. Immunostaining of HCECs Treated with LPS

**Figure 9:**
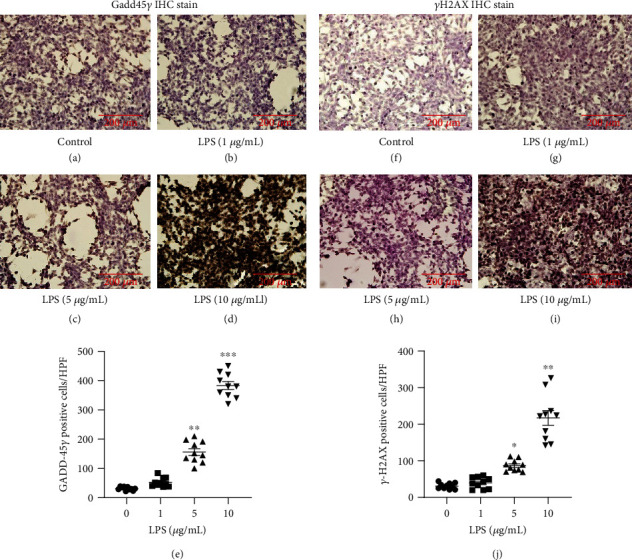
Analysis of growth arrest and DNA damage-inducible factor immunostaining in HCEC cells treated with lipopolysaccharide (1, 5, and 10 *μ*g/mL). An immunohistochemistry stain of Gadd45*γ* showing light microscopic images (a)–(d). To count the Gadd45*γ* positive cells per HPF magnification, densitometry analysis was performed using Image-J software (e). Using immunohistochemistry stains, (f)–(i) show light microscopy images of phosphor-*γ*H2AX. The (j) graph shows the densitometry analysis of cells containing positive *γ*H2AX using Image-J software to quantify the number of positive cells. Based on three cultures replicated for each experimental points (*n* = 6), the data represent Mean ± Standard Error (S.E.). *P* values of 0.05, 0.01, and 0.001 representing ∗, ∗∗, and ∗∗∗ as compared to untreated control cells (NC) were calculated using Kruskal-Wallis and Dunn's tests. The images were all captured at a 20× magnification.

Immunohistochemistry (Gadd45*γ* and *γ*-H2AX) was performed on HCECs treated with LPS (1, 5, and 10 *μ*g/mL) and untreated control cells (Figure 9). The number of Gadd45*γ* antibody-stained positive cells did not significantly differ between the untreated HCECs and HCECs treated with 1 *μ*g/mL LPS (Figures 9(a) and 9(b)). In contrast, treated HCECs with 5 and 10 *μ*g/mL LPS showed an increase of Gadd45*γ* stained positive HCECs (156.2 ± 8.34 and 383.34 ± 14.65, respectively) compared to untreated control cells (Figures 9(c) and 9(d)). The graph Figure 9(e) displays quantitative analytical data of Gadd45*γ* stained positive HCECs. Figures 9(f) and 9(g) show in untreated controls and HCECs treated with 1 *μ*g/mL LPS, the number of *γ*H2AX antibody-stained positive cells did not change significantly (Figures 9(f) and 9(g)). It is shown in Figures 9(f) and 9(g) that the number of *γ*H2AX antibody-stained positive cells/HPF was increased significantly upon treatment with 5 or 10 *μ*g/mL LPS (86.9 ± 5.24 and 217.1 ± 10.52, respectively) compared with the untreated control HCECs (Figures 9(h) and 9(i)). The graph Figure 9(j) displays quantitative data of *γ*H2AX stained positive HCECs.

## 4. Discussion

Current ocular toxicology techniques are not adequate to meet modern needs. It is essential that the assays were able to predict short-term effects on genotoxicity, mutagenicity, and DNA damage. Through an *in vitro* assay that assesses changes in biological activity using primary cells, cell lines, or cellular components, preferably from healthy human donors, high-throughput screening assays have allowed us to diagnose ocular toxicity and corneal dystrophy [31, 32]. Microbial keratitis of corneal dystrophy, however, remains a clinical challenge, with around 50% of patients having poor visual outcomes. Thus, the present study investigated how LPS-induced genotoxicity in HCECs was affected in a time and dose-dependent manner.

A long-term exposure to an endotoxin like LPS may result in the inhibition of cell growth and enhanced mutation and apoptosis through DNA damage and repair byproducts. In fact, oxidative DNA damage caused by endotoxins and cytotoxic drugs can also be mediated primarily by ROS [33]. Furthermore, oxidative stress and inflammation are typically associated with LPS-induced ROS generation, which affects DNA and protein stability, resulting in increased collagen and biomolecule destruction [34]. The activation of nuclear transcription factors by ROS may also enhance the activation of oxidative stress-inducible nuclear transcription factors, which can lead to the overexpression of NF-*κ*B as well as the transcription of proinflammatory responses and DNA strand breaks [35]. A number of cellular mechanisms sense and repair DNA damage, including DNA damage checkpoint, DNA damage repair, and DNA damage tolerance pathways that are critical for cellular stability and regeneration [36]. The findings suggest that ROS production and membrane depolarization of corneal epithelial cells lead to DNA damage and inflammation by causing genotoxic stress-mediated apoptosis. ROS signals have been shown to influence genomic instability and nuclear transcriptional factors to approach apoptotic induction in HCECs, but the function of these factors has not been elucidated yet.

According to previous research, NF-*κ*B functioned broadly to coordinate cellular responses during inflammation [37]. It can be activated by DNA damage as well as most signal transduction pathways that trigger activation of cytoplasmic NF-*κ*B to stimulate inflammatory response. PCR and immunoblotting results in this study showed significantly increased levels of NF-*κ*B in HCECs treated with LPS. The analyses revealed that NF-*κ*B was expressed as mRNA at 1 and 2 hours and as proteins at 4 and 8 hours. Accordingly, we hypothesized that NF-*κ*B activation is a consequence of LPS-mediated ROS accumulation and increased membrane permeability in HCECs. As a result, cells are frequently exposed to oxidative stress, which activates the translocation of NF-*κ*B from the cytoplasm to the nucleus, modulating downstream gene transcription factors. A surplus of NF-*κ*B increased the inflammatory response induced by LPS, which was associated with the upregulation of GADD45G activity in HCECs.

Moreover, the studies documented that the cytotoxicity and genotoxicity tests are rapid methods for assessing the innocuousness and possible beneficial effects of single molecules or multiple mechanisms [38, 39]. A double-strand break (DSB) in DNA and H2AX, a variant of the H2A family of histones, appears to cause the most severe lesions. As a result of a DNA double-strand break, H2AX omega-4 serine residue is rapidly phosphorylated into *γ*H2AX, an event that requires activation of PI3 kinases such as DNA-protein kinases [40]. It is often found that hundreds or thousands of *γ*H2AX molecules accumulate around a single DSB, forming a focus around the DSB, as a marker of DSBs [41]. Therefore, our present results suggest that oxidative stress-induced DNA double-strand breaks activate *γ*H2AX through NF-*κ*B and GADD45G nuclear transcription factors. In HCECs treated with LPS, *γ*H2AX is an important end products for DNA damage.

Additionally, corneal inflammatory dystrophy is characterized by heightened expression of multiple antibodies, which may bind and activate transcription factors such as NF-*κ*B that acetylate core histones and switch genes on [42–44]. Thus, the present results show that the upregulation of genes involved in DNA damage, such as PARP1, Tp53, MSH2, Ogg1, and BRCA1, upregulates both NF-*κ*B and *γ*H2AX expression and DNA damage. DNA damage end points are regulated by these genes that interact with the specific protein-binding domain of *γ*H2AX. The protein could also regulate the activation of phosphorylated Akt in later stages of development. LPS-treated HCECs showed inhibition of cell growth due to these signals, but were not affected by the Erk pathway. In this study, phosphorylated Akt was one of the additional molecules, so it may contribute to apoptosis and cell death later during LPS treatment in HCECs. The function of Akt and Erk-mediated PI3 protein kinase versus the *γ*H2AX signaling pathway requires further investigation.

According to the early reports, in addition to LPS-mediated inflammatory responses, the corneal epithelium naturally accumulates nonspecific innate immune defense mechanisms of the host, which manifest as several immunological secretary molecules (Ig, TLRs, LBP), mucin, growth factors, and excretory molecules (CD, interleukins, and leukotrienes). Under normal conditions, these molecules act as a physical barrier against infection, cell proliferation, survival, and migration [20]. When corneal epithelial cells are exposed to pathological conditions, LPS glycopeptides bound to epithelium surface, and then excessive amounts of infiltrated immunological secretary molecules and their soluble mediators also exacerbate inflammatory responses. These are another important factor for regulating epithelial cell inflammation and apoptotic cell death; later, it plays a role in epithelial cell degeneration and corneal remodeling.

## 5. Conclusion

As a result, the study evaluated the *in vitro* findings suggesting that Gadd45g can cause DNA damage and histone modification-mediated apoptosis in HCECs treated with LPS, as well as previous reports documenting inflammatory responses triggered by LPS. The increased understanding of the NF-*κ*B-GADD45G-*γ*H2AX signaling cascades in infectious keratitis might offer novel antigenotoxic and anti-inflammatory treatments for corneal dystrophy and epithelial cell remodeling. In HCECs, the role of the *γ*H2AX signaling pathway needs to be explored elaborately.

## Figures and Tables

**Table 1:** Shown the list of primers used for qRT-PCR and RT-PCR gene profiles.

S. no	List of genes	Genome ID	Forward primer (5′ → 3′)	Reverse primer (5′ ← 3′)
1.	GADD45B	NM_006705	TGTACGAGTCGGCCAAGTTG	ATTTGCAGGGCGATGTCATC
2.	GADD45G	NM_006705	CTAGCCGTGGCAGGAGCAGC	TGAGCAGCTTGGCCGCTTCG
3.	PARP1	NM_001618	GGAGTCGATCTTGGA	AGTAATAGGCATCGCTCTTGAAGAC
4.	Tp53	NM_000546	GGGTTAGTTTACAATCAGCCACATT	GGGCCTTGAAGTTAGAGAAAATTCA
5.	ATM	NM_000051	TTCCATACCTGAAGTGTAGCATAAA	AATTTGCCAGTCTCATTAACCC
6.	OGG1	NM_016821	TTCCAAGAGGTGGCTCAGAAAT	CGATGTTGTTGTTGGAGGAACA
7.	OXER1	NM_148962	CCTGCACTTTCACCTTCCCT	CACTTTGATCAACCGCTGCC
8.	SCCA1	NM_006919	CAAAGGGCAGTGGGAGAAGA	CCTTGGCCTGTACATCCTCC
9.	MSH2	NM_000251	GCGTCTAAGGAGAATGAGTGG	ACAAGCCTAGCTTCCTCTGG
10.	BRCA1	NM_007295	CAACATGCCCACAGATCAAC	ATGGAAGCCATTGTCCTCTG
11.	DDB1	NM_001923	ATCATCCGGAATGGAATTGGAA	TCAGACCGCAGTCGCCATAA
12.	XRCC1	NM_006297	GCTCGACTGTCACCGCATG	GAACCTGGCCCTGCCATG
13.	ERCC1	NM_001983	TCTCCCCGGTGACTGAATGT	GCGATGAGCTGTTCCAGAGAT
14.	ERCC2	NM_000400	GGCAAAGTGTCCGAGGGAAT	CCTTGAGAATGCGGCTCTGT
15.	GAPDH	2597	TGCACCAACTGCTTAGC	GGCATGGACTGTGGTCATGAG
16.	GADD45G	RT-PCR (NCBI)	AACTAGCTGCTGGTTGATCG	CGTTCAAGACTTTGGCTGAC
17.	NF-*κ*Bp65	RT-PCR(NCBI)	GCCGTGGAGTACGACAA	CGGTTTCCCATTTAGTATGT
18.	*γ*H2AX	RT-PCR(NCBI)	TGGAAAGGGTCAGGGAACG	GACTTGTGCTGGTATCTGGGTG

**Table 2:** Shown the list of primary antibodies used for Western blot protein expression.

S. no	List of antibodies	Catalogue number and source	Raised in	Molecular weight
1	Akt	mAb2920, cell signaling technology	Mouse	60 kDa
2	Phospho-Akt	mAb4051, cell signaling technology	Mouse	60 kDa
3	Erk	mAb4695, cell signaling technology	Mouse	42, 44 kDa
4	Phospho-Erk	mAb4376, cell signaling technology	Mouse	42, 44 kDa
5	Anti-Gadd45*γ*	Ab140378, Abcam	Mouse	17 kDa
6	Phosphor-NF-*κ*B (p65)	mAb3033T, cell signaling technology	Mouse	65 kDa
7	Gamma-H2AX	NB100-384, Novus biological	Rabbit	15 kDa
8	*β*-Actin	mAb3700, cell signaling technology	Mouse	45 kDa

## Data Availability

The data used to support the findings of this study are included within the article.
